# Light-sheet microscopy reveals dorsoventral asymmetric membrane dynamics of *Amoeba proteus* during pressure-driven locomotion

**DOI:** 10.1242/bio.059671

**Published:** 2023-02-23

**Authors:** Atsushi Taniguchi, Yukinori Nishigami, Hiroko Kajiura-Kobayashi, Daisuke Takao, Daisuke Tamaoki, Toshiyuki Nakagaki, Shigenori Nonaka, Seiji Sonobe

**Affiliations:** ^1^ Laboratory for Spatiotemporal Regulations, National Institute for Basic Biology, Okazaki, Aichi, Japan; ^2^ Spatiotemporal Regulations 444-8585 Group, Exploratory Research Center on Life and Living Systems (ExCELLS), Okazaki, Aichi 444-8585, Japan; ^3^ Research Institute for Electronic Science, Hokkaido University, Sapporo 001-0020, Japan; ^4^ Laboratory of Regeneration Biology, National Institute for Basic Biology, Okazaki, Aichi 444-8585, Japan; ^5^ Graduate School of Medicine, The University of Tokyo, Bunkyo, Tokyo 113-0033, Japan; ^6^ Faculty of Science, Academic Assembly, University of Toyama, Gofuku, Toyama 930-8555, Japan; ^7^ Graduate School of Life Science, University of Hyogo, Kamigori, Hyogo 678-1297, Japan

**Keywords:** Amoeboid locomotion, Cell locomotion, Light sheet microscopy, Membrane dynamics

## Abstract

Amoebae are found all around the world and play an essential role in the carbon cycle in the environment. Therefore, the behavior of amoebae is a crucial factor when considering the global environment. Amoebae change their distribution through amoeboid locomotion, which are classified into several modes. In the pressure-driven mode, intracellular hydrostatic pressure generated by the contraction of cellular cortex actomyosin causes the pseudopod to extend. During amoeboid locomotion, the cellular surface exhibits dynamic deformation. Therefore, to understand the mechanism of amoeboid locomotion, it is important to characterize cellular membrane dynamics. Here, to clarify membrane dynamics during pressure-driven amoeboid locomotion, we developed a polkadot membrane staining method and performed light-sheet microscopy in *Amoeba proteus*, which exhibits typical pressure-driven amoeboid locomotion. It was observed that the whole cell membrane moved in the direction of movement, and the dorsal cell membrane in the posterior part of the cell moved more slowly than the other membrane. In addition, membrane complexity varied depending on the focused characteristic size of the membrane structure, and in general, the dorsal side was more complex than the ventral side. In summary, the membrane dynamics of *Amoeba proteus* during pressure-driven locomotion are asymmetric between the dorsal and ventral sides.

This article has an associated interview with the co-first authors of the paper.

## Teaser

**Summary:** *Amoeba proteus* exhibits dorsoventral asymmetric membrane dynamics during pressure-driven amoeboid locomotion.

## INTRODUCTION

Amoebae are a group of protists adapted to diverse environments on Earth and can be found in freshwater ponds, swamps, brackish water, oceans and soil (Anderson, 2017). In freshwater, amoebae are major predators of microorganisms (e.g. bacteria, algae, and ciliates). Amoebae are involved in the carbon cycle by performing the remineralization of nutrients through predation. Recent studies suggest that amoebae suppress increases in bacteria in biofilms (Anderson, 2013; Zhang et al., 2014). In addition, amoebae promote plant growth through the remineralization of nutrients by predation in the rhizosphere (Bonkowski et al., 2001; Bonkowski and Brandt, 2002). Thus, amoebae play an essential role in the global environment (Anderson, 2017). Knowledge of the mechanism of amoeboid movement contributes to understanding the organism's behavior and, ultimately, the environment. Adherent cells generally exhibit amoeboid locomotion, as observed in amoebae and motile mammalian cells (Lämmermann and Sixt, 2009). In this process, cells elongate their pseudopods, and the mechanisms of elongation are categorized into three types: actin-driven, friction-driven, and pressure-driven protrusion (Petrie and Yamada, 2016). Although each cell type exhibits dominant mechanisms, cells switch between different mechanisms depending on the external environment. In actin-driven movement, actin polymerizes at the cell tip and pushes the cell membrane outward to form a pseudopod, which carries out amoeboid movement. Various types of cells show actin-driven movement on a two-dimensional glass surface (i.e. fibroblasts, leukocytes) (Pollard and Borisy, 2003; Mattila and Lappalainen, 2008; Caswell and Zech, 2018). In friction-driven amoeboid movement, the force generated by the retrograde flow of actomyosin is transferred to the outside of the cell via friction between the substrate and the cellular surface. Some nonadherent cells sandwiched between two substrates show friction-driven movement (Bergert et al., 2015; Petrie and Yamada, 2016). Then, during pressure-driven movement, cells generate intracellular hydrostatic pressure by contracting actomyosin at the cell cortex. Intercellular pressure pushes the cell membrane outward, causing the pseudopod to protrude (Fackler and Grosse, 2008; Charras and Paluch, 2008; Petrie and Yamada, 2016). In mammals, primordial germ cells exhibit pressure-driven movements known as blebbing (Blaser et al., 2006), and actin-driven cells, such as fibroblasts and cancer cells, have been reported to show bleb protrusion under certain conditions (Paul et al., 2017). In addition, a kind of protists, *Amoeba proteus,* forms wide, thick pseudopods known as lobopods for locomotion and consistently exhibits pressure-driven amoeboid movement (Mast, 1926; Goldacre, 1961; Nishigami et al., 2013; Matsumoto et al., 2022).

During amoeboid movement, the cellular shape drastically changes. Therefore, measuring cell membrane dynamics during amoeboid movement is necessary to elucidate the mechanism of the movement. In this regard, several relevant experiments have been reported. For example, in *Vannella*, an amoeba with actin-driven protrusion, a dorsally attached marker moves in the same direction as the cell. When the marker reaches the anterior end, it shifts to the ventral side and remains there until it reaches the posterior end. After reaching the dorsal surface, the marker exhibits the same pattern. These results suggested that the *Vannella* membrane moves in a caterpillar-like rotation (Komnick et al., 1973). Photobleaching experiments performed on polymorphonuclear leukocytes treated with membrane staining reagents that reveal actin-driven pseudopodia revealed that dorsal and ventral cell membranes move in the direction of cell motility at the same rate (Lee et al., 1990). Similar experiments have been performed in *Dictyostelium discoideum*, in which both dorsal and ventral cell membranes were observed to be stationary relative to the substrate during cell motility (Tanaka et al., 2017).

Although the results are varied, there are many reports of the membrane dynamics of actin-driven locomotion. Regarding the membrane dynamics of cells moving under pressure-driven conditions, there have been reports in *A. proteus* involving markers such as microbeads and activated charcoal (Griffin and Allen, 1960; Haberey et al., 1969; Komnick et al., 1973; Grebecki, 1986). These studies have reported that markers attached to the sides and front of the pseudopod move in the direction of cell movement. They have also shown that markers attached to wrinkles in the posterior region of the cell stop when they contact the substrate and move forward on the cell when the wrinkles unfold. These results proposed a model, called total folding and unfolding (TFU) (Czarska and Grebecki, 1966; Stockem et al., 1969; Haberey et al., 1969; Komnick et al., 1973). In the TFU model, cells use wrinkles of cellular membranes for amoeboid locomotion. In the posterior region of the cell, cells create wrinkles by contracting the cell cortex. Simultaneously, they unfold the wrinkles of the plasma membrane to extend the pseudopods in the area between the anterior and posterior regions of the cell. The TFU model focuses on the difference between the cell's anterior and posterior regions; the cell's ventral and dorsal sides are considered equivalent. However, the membrane dynamics on the ventral side have not yet been observed during pressure-driven amoeboid movement. Therefore, we attempted to visualize cell membrane dynamics, including those on the ventral side, using *A. proteus*, which has long been studied as an experimental organism and consistently exhibits pressure-driven amoeboid movement. Light-sheet microscopy has been demonstrated to capture the rapid movement of amoebae (Takao et al., 2012). In light-sheet microscopy, it is possible to change the position and angle of the specimens relative to the excitation sheet light, which makes it easy to obtain the sagittal plane images. Therefore, the observation method is suitable for clarifying differences in cell membrane dynamics between the dorsal and ventral regions (Huisken et al., 2004). In addition to this microscopy technique, we have developed a method for staining membranes in a dotted pattern. By using these methods, we succeeded in capturing the membrane dynamics of *A. proteus*.

## RESULTS

### Membrane visualization

**Fig. 1.**
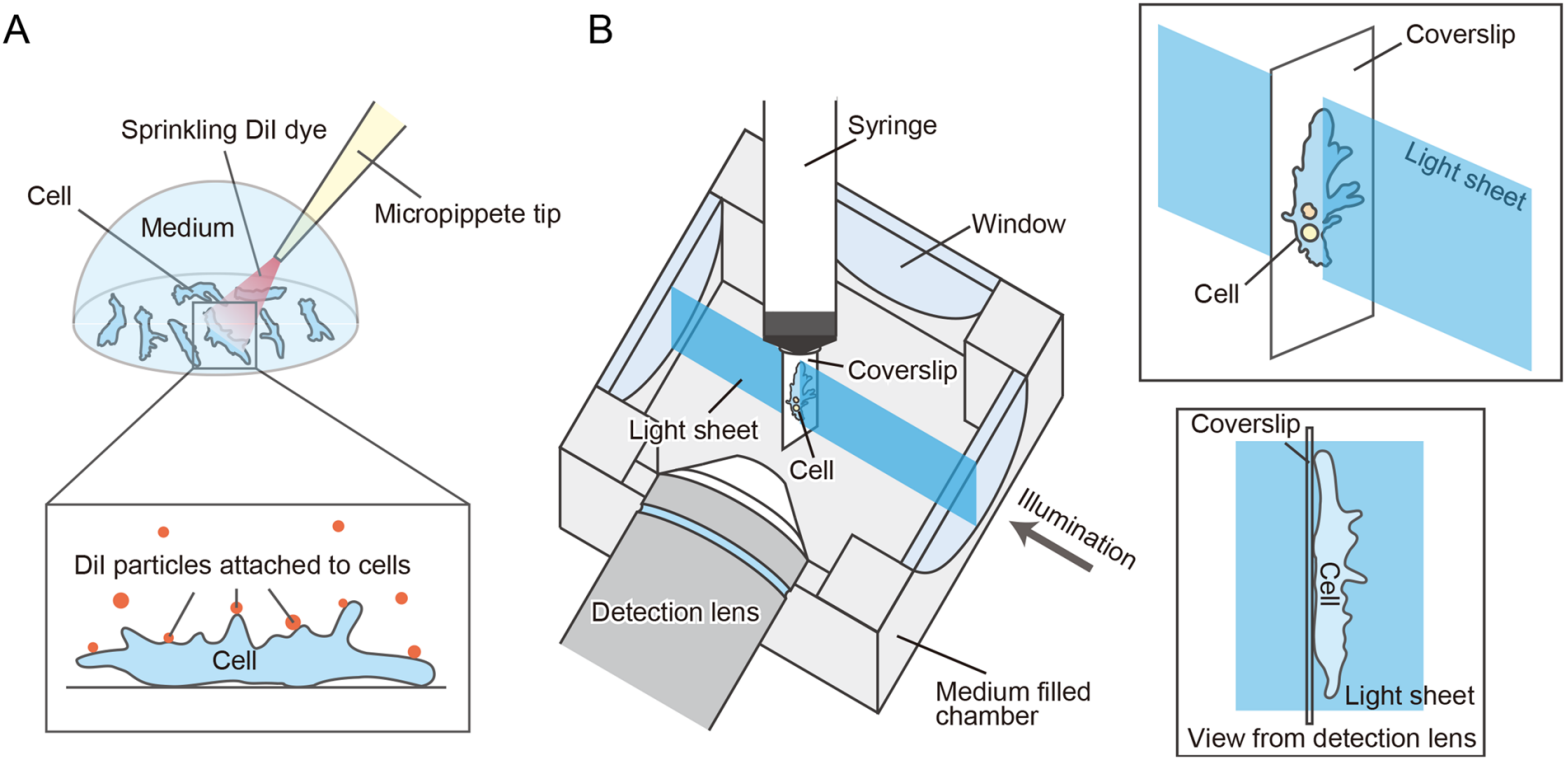
**Visualization of the membrane dynamics of *A. proteus*.** (A) A schematic diagram of the DiI dot staining of *A. proteus*. By spraying DiI onto the cells with a micropipette, DiI dots were deposited on the cell surface. By using the dots as markers, it was possible to track membrane dynamics. (B) Schematic of a cell mounted in the chamber of the light sheet microscope. The upper right shows that the excitation light sheet irradiation is applied perpendicular to the sample. The lower right shows a schematic of the cell viewed from the detection lens side, indicating that the obtained fluorescence image is in the sagittal plane of the cell.

**Fig. 2.**
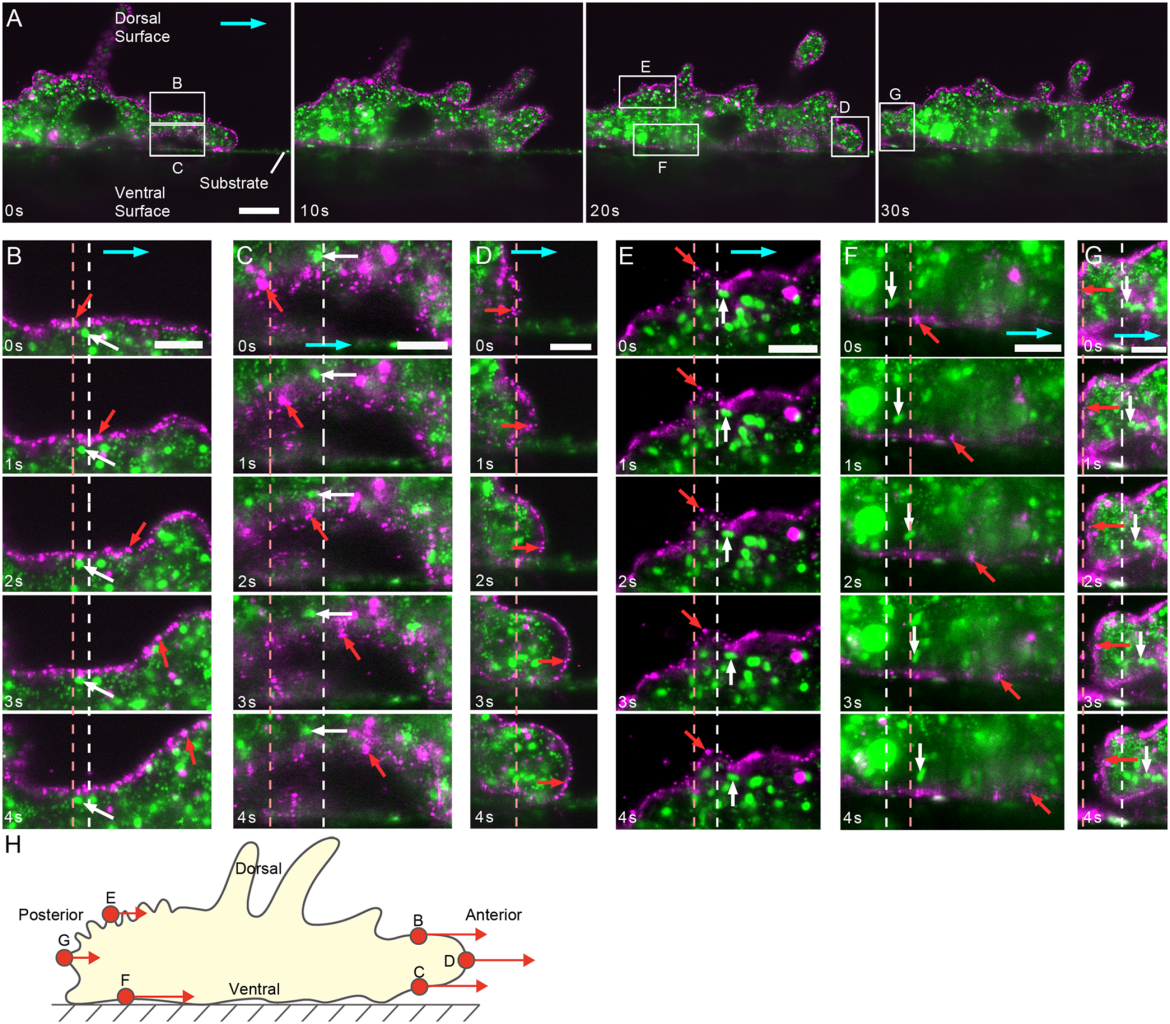
**Cell membrane and cytoplasmic dynamics of *A. proteus* observed by light-sheet microscopy.** (A) A time-lapse image of *A. proteus* observed by light-sheet microscopy, in which the cell membrane (magenta) was stained by DiI and the cytoplasm (green) by MitoTracker. The light blue arrows indicate the direction of cell movement. B-G show magnified images of the regions shown in A. B and C are the dorsal and ventral sides of the anterior part of the cell, E and F are the dorsal and ventral sides of the posterior part of the cell, and D and G are the anterior and posterior ends of the cell. The red arrows indicate the location of DiI attached to the cell membrane, and white arrows indicate the positions of mitochondria in the cytoplasmic gel. The light red dashed lines indicate the positions of the DiI marker at 0 s, and the white dashed lines indicate the mitochondria in the gel at 0 s. (H) The results in B-G are summarized in a schematic. The red circles represent DiI in B-G, and the red arrows indicate the velocity of the DiI particles. Scale bars: 30 µm in A and 10 µm in B-G.

To observe membrane dynamics, it is necessary to mark the cell membrane. In previous reports, membrane dynamics have been observed by attaching markers such as microbeads (Grebecki, 1986; Yumura et al., 2013) or activated charcoal (Griffin and Allen, 1960; Haberey et al., 1969) to the cell membrane or by photobleaching a part of the cell following fluorescent staining of the membrane (Lee et al., 1990; Tanaka et al., 2017). However, in the case of marker attachment, it is unclear whether a marker on the substrate side is merely attached to the substrate or reflects the membrane dynamics on the ventral side. In photobleaching experiments, it is difficult to observe the membrane dynamics of the entire cell. Therefore, we developed a polkadot membrane staining method. Since the membrane-staining dye DiI is lipophilic, it forms particle-like clusters in an aqueous solution. After DiI was sprayed on *A. proteus,* DiI particles adhered to or partially stained the cell membrane, which served as a marker to visualize membrane dynamics (Fig. 1A). Since the entire cell could be stained with dots, it was possible to visualize the membrane dynamics of the entire cell. In this experiment, we tried to observe the membrane dynamics of the ventral and dorsal sides of the cell simultaneously (Fig. 2).

### Cell membrane and cytoplasmic dynamics

**Fig. 3.**
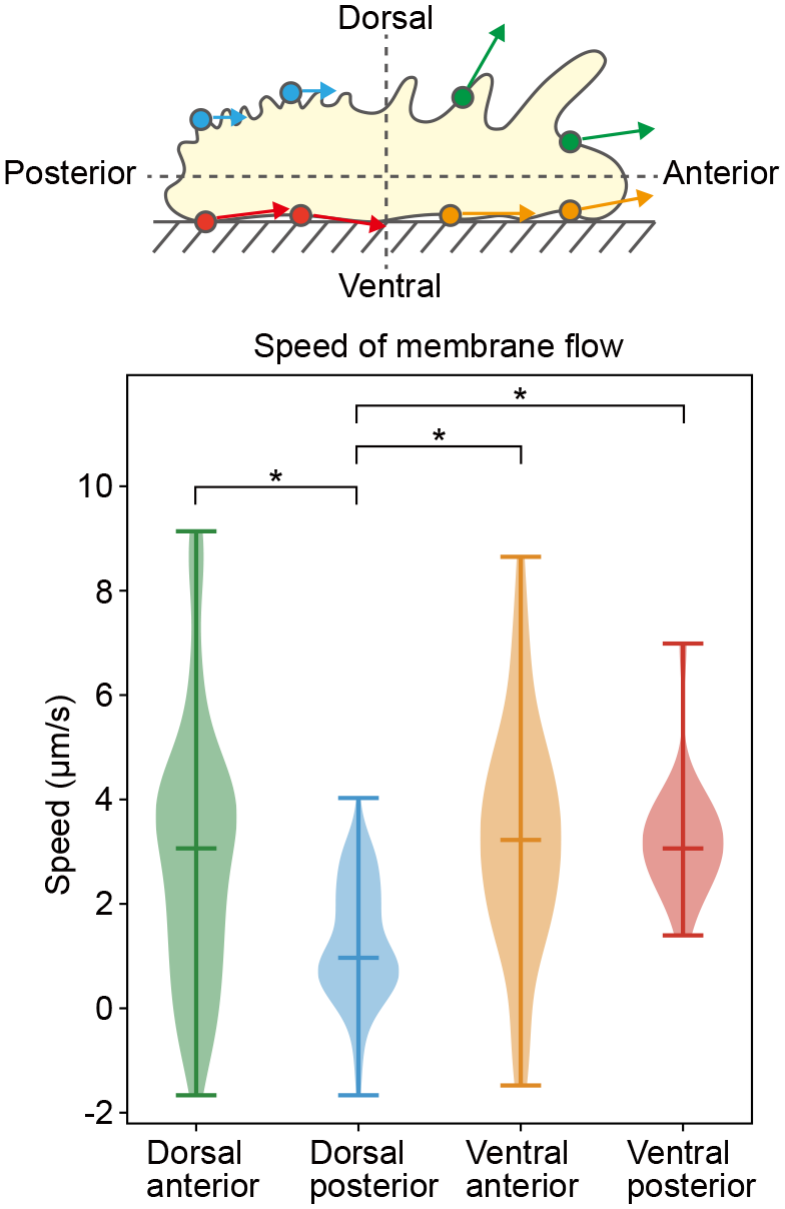
**The speed of the plasma membrane at each position.** The upper image shows a schematic diagram of the analysis of the speed of movement. In the analysis, the cell was divided into four regions: dorsal anterior, dorsal posterior, ventral anterior, and ventral posterior. The lower figure shows the values of the speed in each group. In the dorsal posterior region, the speed of the membrane was slower than that in other parts of the body (*n*=194, Welch's *t*-test, *P<0.01).

We used a digital scanned laser light-sheet fluorescence microscope (DSLM) to observe *A. proteus* membranes labeled with DiI as described above. The movement of *A. proteus* is fast (1 µm/s) making it difficult to obtain three-dimensional data. Therefore, we irradiated the cells with a sheet of excitation light from the dorsal view, perpendicular to the cells, as shown in Fig. 1B, and acquired fluorescent images in the sagittal plane. Using this observation method, we could observe the dorsal and ventral membrane dynamics simultaneously. *A. proteus* exhibits active cytoplasmic flow during amoeboid movement (Mast, 1926). Therefore, to visualize the dynamics of the cytoplasm, we stained *A. proteus* with MitoTracker, a mitochondrial staining reagent. Fig. 2A-G and Movies 1-7 show time-lapse images of *A. proteus* undergoing amoeboid locomotion, with the plasma membrane shown in magenta and mitochondria in green. In the images, the cells are moving to the right, and all regions of the cell membrane (anterior, posterior, dorsal, and ventral) generally move in the direction of cell movement (tangential to the membrane), although the speed of movement varies. The membrane at the tips of the cell moved in the direction perpendicular to the membrane, which was also the direction of cell motion (Fig. 2D,G; Movies 4 and 7). The speed of the dorsal posterior side of the cell was significantly slower than that of the rest of the cell (Fig. 2E and Movie 5). We categorized the parts of cells into four areas: dorsal anterior, ventral anterior, dorsal posterior, and ventral posterior, and the speed of the membrane was measured (Fig. 3). The results showed that the dorsal anterior cell membrane moved at 3.06±0.18 (median±s.e.m) µm/s, the dorsal posterior at 0.97±0.09 µm/s, the ventral anterior at 3.22±0.24 µm/s, and the ventral posterior at 3.06±0.27 µm/s. Since mitochondria are trapped by intracellular structures such as actin fibers in *A. proteus* (Stockem et al., 1982; Jeon, 1973), we assumed that mitochondrial movement was closely related to the cytoplasmic flow. When we observed the mitochondria undergoing amoeboid movement, we found that even when the plasma membrane was moving in the direction of cell movement, the mitochondria were almost stationary in relation to the substrate near the plasma membrane, regardless of whether they were on the ventral or dorsal side (Fig. 2B,C,E-G and Movies 2, 3, 5-7). On the other hand, the mitochondria away from the cell membrane moved at high speed (Movie 1), which was difficult to evaluate quantitatively.

### Cell membrane structure complexity

**Fig. 4.**
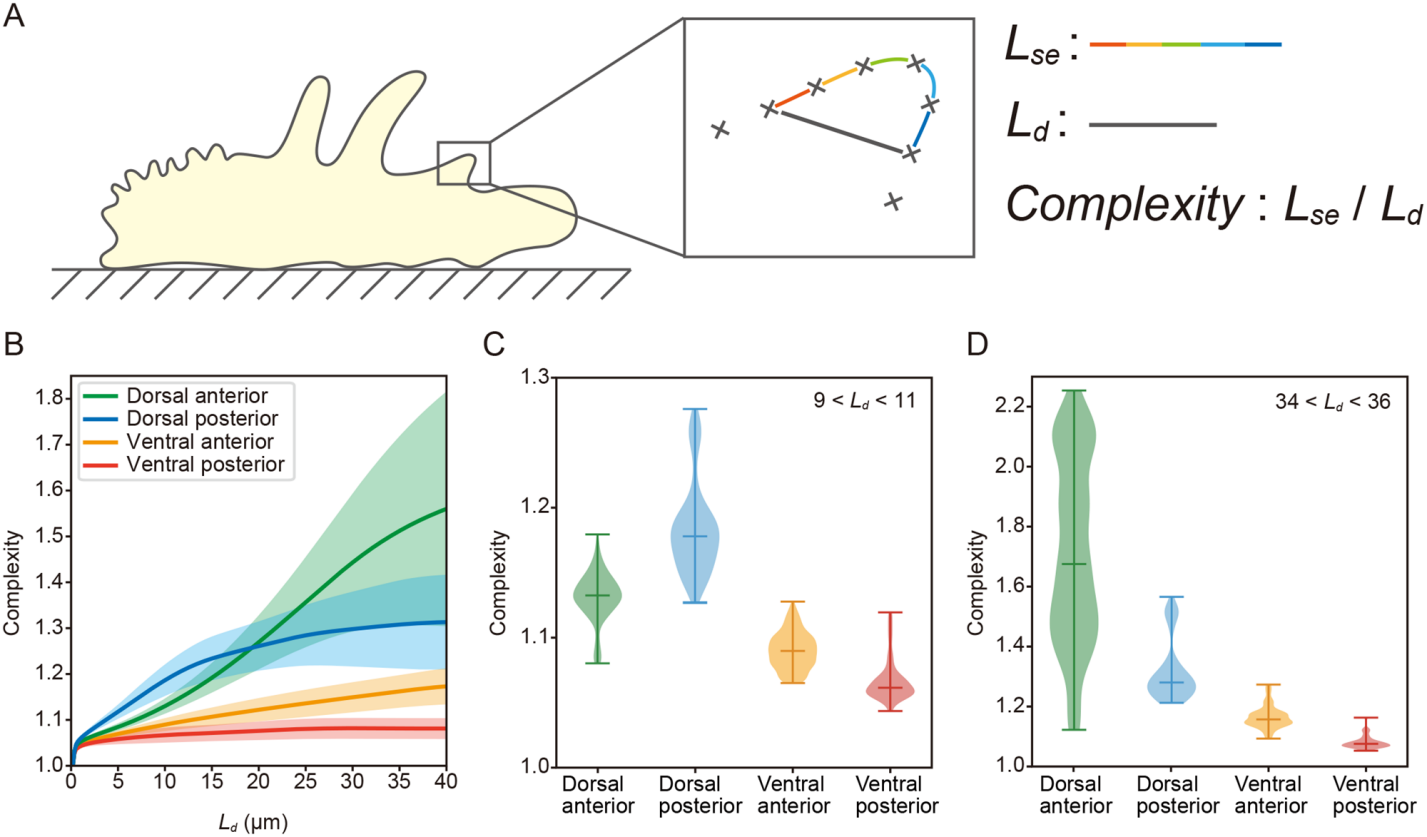
**Complexity of membrane shape at each position.** (A) We extracted the coordinates of the cell membrane and calculated the complexity. The definition of complexity for an interval is the sum of the distances between each point in that interval (*L*_*se*_) divided by the length of direct connections (*L*_*d*_). (B) Plot of complexity versus the length of direct connections (*L*_*d*_) in each region of the cell membrane (*n*=32). The solid line shows the mean complexity, and the transparent area shows the population standard deviation. Regardless of *L*_*d*_, the ventral side was simpler than the dorsal side, and the ventral anterior was more complex than the ventral posterior. On the other hand, for the dorsal side, the relationship between the magnitude of complexity of the anterior and posterior portions varied with the value of *L*_*d*_. For small *L*_*d*_, the dorsal posterior was more complex than the anterior (violin plot shown in C), and for large *L*_*d*_, the dorsal anterior was more complex than the posterior (violin plot shown in D). Note that all values shown in C were significantly different from each other (*n*=32, Welch's *t*-test, *P<0.01). All values in D also differed significantly from each other (*n*=32, Welch's *t*-test, *P<0.01).

To understand the mechanism underlying the site-dependent differences in membrane dynamics, we quantitatively evaluated the complexity of the structure of the cell membranes (Fig. 4). Cell membrane positions were extracted from stained images of cell membranes in the sagittal plane and classified according to their positions. As shown in Fig. 4A, we measured the length of the direct connection between two focused points on the cell membrane (*L*_*d*_) and the sum of the distances between each point between the focused points (*L*_*se*_). We defined a value, *L*_*se*_ divided by *L*_*d*_, as a complexity (details in Materials and Methods and Fig. S1). The larger this value is, the higher the complexity. In Fig. 4B, we plot the relationship between complexity and the length of the direct connection between the two focused points (*L*_*d*_). Regardless of *L*_*d*_, the ventral posterior was the simplest, followed by the ventral anterior. On the dorsal side, the dorsal posterior was more complex than the dorsal anterior, when *L*_*d*_ was approximately less than 19 µm. On the other hand, the dorsal anterior was more complex than the dorsal posterior when *L*_*d*_was larger than approximately 19 µm. Fig. 4C shows each cellular region's complexity at 9<*L*_*d*_<11. The results showed that the complexity of the cell membrane was 1.13±0.02 (median±s.d.) at the dorsal anterior, 1.18±0.04 at the dorsal posterior, 1.09±0.01 at the ventral anterior, and 1.06±0.02 at the ventral posterior. By Welch's *t*-test, all these results were significantly different from each other (*P*<0.01). Then, for large *L*_*d*_ between 34<*L*_*d*_<36, the complexity of the cell membrane was 1.68±0.31 (median±s.d.) at the dorsal anterior, 1.28±0.11 at the dorsal posterior, 1.16±0.04 at the ventral anterior, and 1.08±0.02 at the ventral posterior. By Welch's *t*-test, all these results were significantly different from each other (*P*<0.01).

## DISCUSSION

Amoebae inhabit various environments and are essential organisms involved in carbon cycling in the global environment because they act as significant predators of microorganisms at the bottom of the environmental waters. In this context, the behavior of this group of organisms may have a substantial impact on the global environment. To clarify the details of this behavior, we focused on the dynamics of the cell membrane in the amoeboid movement of *A. proteus*, which has long been studied as a model cell of amoeboid movement.

*A. proteus* is thought to move via the creation of intracellular pressure by the contractile force of the actomyosin system on the cell surface (Mast, 1926; Goldacre, 1961; Nishigami et al., 2013), termed pressure-driven amoeboid locomotion (Petrie and Yamada, 2016). At the pseudopod elongation site, actin on the surface of the cell membrane collapses (Charras and Paluch, 2008), causing cytoplasm to flow into the structurally weakened area driven by intracellular pressure. This inflow of cytoplasm then pushes the cell membrane outward, which is thought to move the pseudopod in the direction of cell movement. In addition to this movement, actomyosin contraction at the cell surface causes the plasma membrane to develop a wrinkled structure, which moves the cytoplasm to the anterior end of the cell and pulls the plasma membrane in the direction of cell movement.

**Fig. 5.**
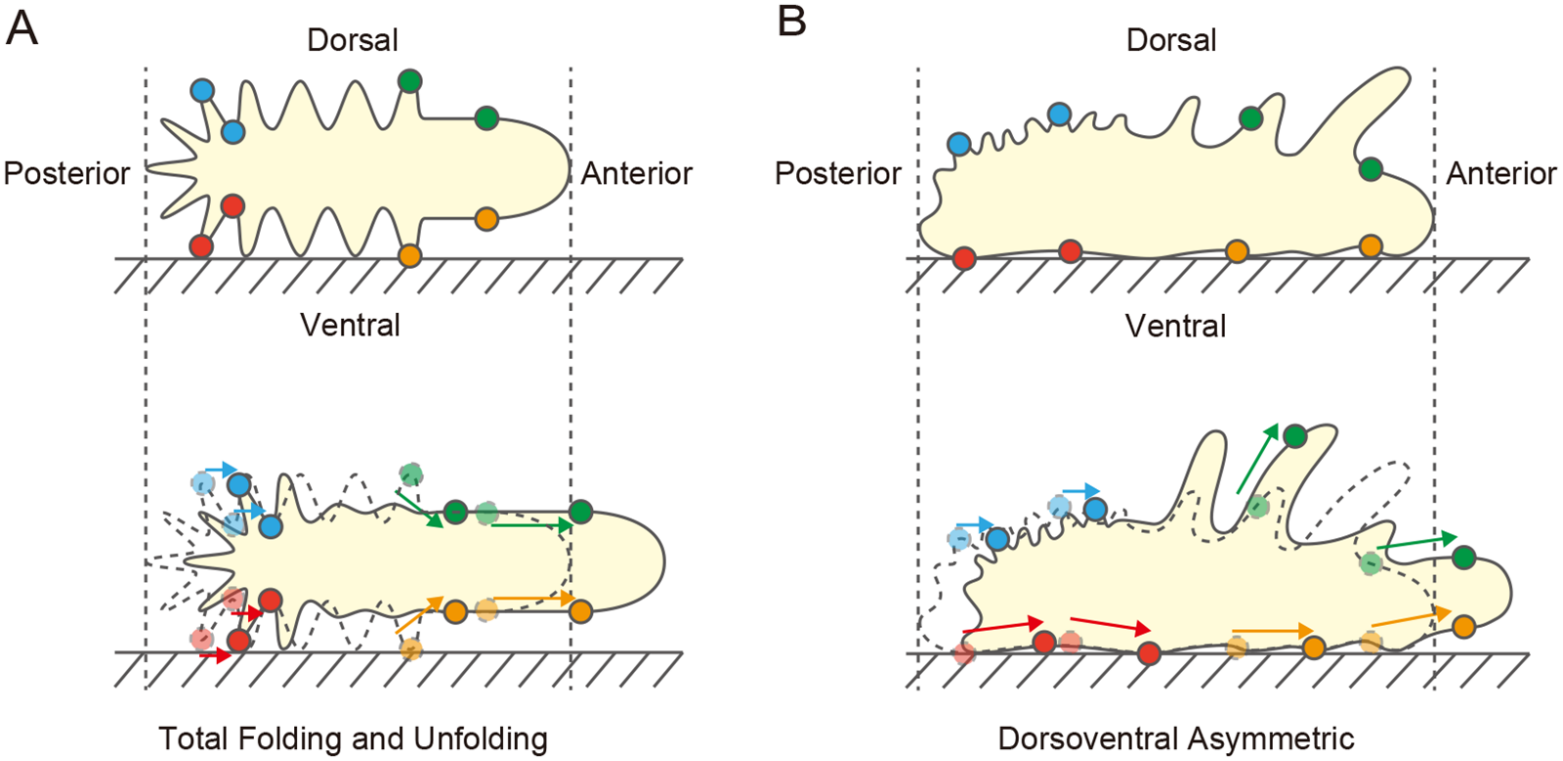
**Schematic side view of the membrane dynamics model in pressure-driven amoeboid locomotion.** (A) TFU model. In this model, wrinkle formation of the cell membrane occurs in the posterior part of the cell, while wrinkles unfold in the central part of the cell. These processes make all cell membranes move in the direction of movement, and the speed of movement is faster in the anterior part of the cell than in the posterior part. The plasma membrane complexity is simpler in the anterior part than in the posterior part. In this model, the dorsal and ventral sides are considered symmetric. (B) In our study, we found that the speed of plasma membrane movement was significantly lower in the posterior dorsal region than in the other regions. We also showed that the complexity of the plasma membrane is lower on the ventral side than on the dorsal side. On the ventral side, larger structures dominated in the anterior region, and smaller ones dominated in the posterior region. These experiments suggest that wrinkle formation occurs in the dorsal posterior region of the plasma membrane and that wrinkle unfolding occurs between the dorsal anterior and posterior regions and between the dorsal and ventral regions of the posterior end of the cell. Thus, there is a dorsoventral asymmetry in membrane motion in pressure-driven amoeboid locomotion.

Then, we focused on membrane dynamics during pressure-driven amoeboid locomotion. Previous studies, in which membrane motion was traced by attaching beads or activated charcoal to the cell surface, have shown that the membrane moves in the forward direction in the anterior and posterior regions of the cell; the speed of membrane flow is faster in anterior than in posterior regions of the cell (Schaeffer, 1920; Mast, 1926; Griffin and Allen, 1960; Haberey et al., 1969; Komnick et al., 1973; Grebecki, 1986). In addition, optical and electron microscopic observations have shown that the plasma membrane forms a wrinkled structure at the posterior region of the cell (Czarska and Grebecki, 1966; Stockem et al., 1969; Haberey et al., 1969; Haberey, 1971; Stockem, 1972). Based on these observations, the total folding and unfolding (TFU) model was proposed in which membrane wrinkle formation occurs in the posterior region of the cell, and the wrinkles unfold in a region between the anterior and posterior regions of the cell (Fig. 5A; Czarska and Grebecki, 1966; Stockem et al., 1969; Haberey et al., 1969; Komnick et al., 1973; Grebecki, 1986). The TFU model focuses positions on the front-rear axis of the cell, and it has been believed that the dorsal and ventral sides behave identically. Because of technical difficulties, previous studies have not examined the uniformity of the membrane dynamics on the dorsal and ventral sides. In this study, we succeeded in simultaneous visualization of dorsal and ventral membrane dynamics and found dorsoventral asymmetric dynamics. Although all membrane flow directions were equal to the direction of motion, which is consistent with the TFU model, the flow speed of the dorsal posterior region was slower than that of the other region of the cell. A possible reason for the dorsoventral asymmetry in flow speed is that winkle formation of the cell membrane could be active in the dorsal posterior region (Fig. 5B). The unfolding of cell membrane wrinkles could occur near the anterior-posterior boundaries of the dorsal side and near the dorsal-ventral boundaries of the posterior end of the cell.

The complexity of the cellular shape varied with *L*_*d*_. In the range where *L*_*d*_ was less than 19 μm, the complexity increased in the order of ventral posterior, ventral anterior, dorsal anterior, and dorsal posterior. The fact that the dorsal posterior region exhibited the most complex shape is consistent with the prediction that wrinkle unfolding of the cell membrane occurs near the dorsal anterior–posterior boundaries and the dorsal–ventral boundary at the posterior end. This fact is also consistent with the prediction that wrinkle formation occurs throughout the dorsal posterior region. As a reason for the increased complexity of the ventral posterior, ventral anterior, and dorsal anterior regions, it is considered that active pseudopod formation occurs in the anterior region, increasing in complexity derived from the pseudopodia structure. Furthermore, since most pseudopodia are formed in the dorsal anterior region, the complexity of the dorsal anterior region tends to be higher. Next, when *L*_*d*_ was greater than 19 μm, complexity increased in the order of ventral posterior, ventral anterior, dorsal posterior, and dorsal anterior. This result differs from the low *L*_*d*_ case in that the dorsal anterior region is more complex than the dorsal posterior region. The magnitude of *L*_*d*_ can be considered a characteristic size of the structure. Therefore, the characteristic sizes of the structures are smaller than 19 µm in the dorsal posterior region, and the characteristic sizes are larger than 19 µm in the dorsal anterior region. The actual structure shows that the dorsal posterior region has more small structures than the dorsal anterior region. Therefore, the difference in complexity of the *L*_*d*_ can be considered to reflect the wrinkle structure and pseudopodia structure. These results indicate that the dorsoventral symmetry previously assumed in the TFU model (Czarska and Grebecki, 1966; Stockem et al., 1969; Haberey et al., 1969; Komnick et al., 1973; Grebecki, 1986) is not valid for pressure-driven amoeboid locomotion, at least in *A. proteus*.

In summary, the membrane dynamics of *A. proteus* during pressure-driven amoeboid locomotion have been considered a dorsoventral symmetric locomotion mechanism (TFU model). However, our results indicate that the cell membrane exhibits dorsoventral asymmetric membrane dynamics. Future studies will be required to verify the generality of our findings by clarifying membrane dynamics during pressure-driven amoeboid locomotion in various cells using the polkadot staining method we have developed.

## MATERIALS AND METHODS

### Cell culture

*Amoeba proteus* was cultured in plastic boxes (300 mm×220 mm×50 mm) filled with KCM medium (9.39 µM KCl, 32.5 µM MgSO_4_·7H_2_O, 54.4 µM CaCl_2_·2H_2_O) at 25°C and fed *Tetrahymena pyriformis* twice a week. *T. pyriformis* was cultured in 2% (w/v) Bacto Proteose Peptone medium (Becton, Dickinson and Company, USA) for 3 days. *A. proteus* was starved for at least 3 days before the experiments.

### Digital scanned light-sheet microscopy

For the simultaneous observation of dorsal and ventral cell surfaces, we used a digital scanned light-sheet microscope (DSLM) at the National Institute for Basic Biology, designed according to the system located at the European Molecular Biology Laboratory (EMBL) (Keller et al., 2008). The DSLM was equipped with continuous wave laser (35 LTL 835-200, Ar-Kr laser, Melles Griot, USA), emission filters (RazorEdge Long Pass 568 and BrightLine Fluorescence Filter 609/54, Semrock, USA), a Plan-Apochromat 5×/0.16 (Zeiss, Germany) illumination lens, a W Achroplan 40×/0.80 (Zeiss) detection lens, and a CCD camera (ORCA-AG; Hamamatsu Photonics, Japan). The image acquisition speed was one FPS. This imaging speed is the maximum speed that can be achieved using the software developed by EMBL. To control the direction of locomoting *A. proteus*, a syringe was used to place a cell extract of homogenized *T. pyriformis* on the cover slip, and the extract was air-dried. *A. proteus* was then placed on this cover slip, which was mounted in the chamber of the DSLM.

### Staining of the cell surface and mitochondria

The cell surface of *A. proteus* was stained with 2.68 µM 1,1′-dioctadecyl-3,3,3′,3′-tetramethylindo-carbocyanine perchlorate (DiI) (ThermoFisher Scientific, USA) sprinkled in fresh medium for 10 min, and the cells were washed three times with fresh medium. The cells were further stained with 0.92 µM MitoTracker Deep Red FM (ThermoFisher Scientific) for 5 min and then washed three times with fresh medium.

### Analysis of cell membrane dynamics

To quantify cell membrane dynamics, we analyzed the images of DiI fluorescent dot movement recorded by the DSLM. For this analysis, cells whose direction of motion was same as the light sheet plane were used. First, the position of a Dil particle was determined based on fluorescence intensity using the image analysis software Fiji (Schindelin et al., 2012) with a particle tracking plugin (MTrackJ; Meijering et al., 2012). Since the temporal resolution of the obtained data was not sufficient for automatic single particle tracking, we manually tracked groups of particles with an unambiguously recognizable pattern in successive frames. In this process, we considered the distribution pattern of particles around the focused particle and treated a particle at a different time with similar patterns around it as the same particle. Even if the pattern changes significantly over time, if we could trace the trajectory of the particle from at least 3 s ago and estimate the location of the particle from the speed and direction of movement in the prior frames, we considered the estimated location was the location of the particle of interest. For the calculation of speed in the direction of the axis of movement, we used the position of the same particle 1 s later. We assumed that the direction of cell movement was positive. This analysis was performed for all of the traceable Dil dots on the dorsal and ventral membrane surfaces. In addition, the positions of the Dil dots were classified into four categories (dorsal anterior, ventral anterior, dorsal posterior, and ventral posterior). Three typical specimens were used for the analysis, and the total number of measurement points was 194. Welch's *t*-test was used to test for significant differences. Python with NumPy (Harris et al., 2020) and SciPy (Virtanen et al., 2020) was used for computation and statistical analysis.

### Analysis of cell membrane structure complexity

When the cell membrane is stained in a polkadot pattern, the whole cell membrane is lightly stained. Therefore, it is possible to extract cell morphology from these images. The cell shapes were binarized according to intensity, and edge detection was performed using the Sobel filter. The coordinates of the edge were considered the coordinates of the cell membrane. We evaluated the complexity of the cell membrane using the positions. First, as in the measurement of the cell membrane speed above, the cell membrane positions were classified into four categories (dorsal anterior, ventral anterior, dorsal posterior, and ventral posterior). In the categorization, the positions at a distance of 15 µm from the edge of the cell were removed because it was difficult to accurately evaluate positions near the dorsal and ventral membrane junctions. As shown in Fig. S1, we define the positional vector on the *i*-th specimen at position number *n*′ as ***p***_*i*_=(***x***_*i*_(*n*′), ***y***_*i*_(*n*′) ). Then, the length between positions ***p***_*i*_(*n*′) and ***p***_*i*_(*n*′+1) is expressed as *L*_*ei*_(*n*′)=|***p***_*i*_(*n*′+1)−***p***_*i*_(*n*′)|. The distance directly connected from ***p***_*i*_(*n*′) to ***p***_*i*_(*n*′+*n*), which is n points ahead, is *L*_*di*_(*n*, *n*′)=|***p***_*i*_(*n*′+*n*)−***p***_*i*_(*n*′)|. On the other hand, the same distance obtained by summing each distance to one neighboring point is 
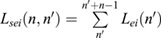
. We define the complexity of the cell as 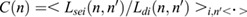

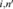
means the ensemble average of the sample and position number. This value is larger for more complex the cell membrane shapes. This analysis was performed on four typical samples and 32 shapes of three cells. Welch's *t*-test was used to test for significant differences. Python with NumPy (Harris et al., 2020) and SciPy (Virtanen et al., 2020) was used for computation and statistical analysis.

## Supplementary Material

10.1242/biolopen.059671_sup1 Supplementary information
