# Supplementary material for: Light-sheet microscopy reveals dorsoventral asymmetric membrane dynamics of Amoeba proteus during pressure-driven locomotion
Source: Biol Open. 2023 Feb 23;12(2):bio059671. doi: 10.1242/bio.059671 (PMC9986612; doi:10.1242/bio.059671)
Supplement: Supplementary information [file biolopen-12-059671-s1.pdf]

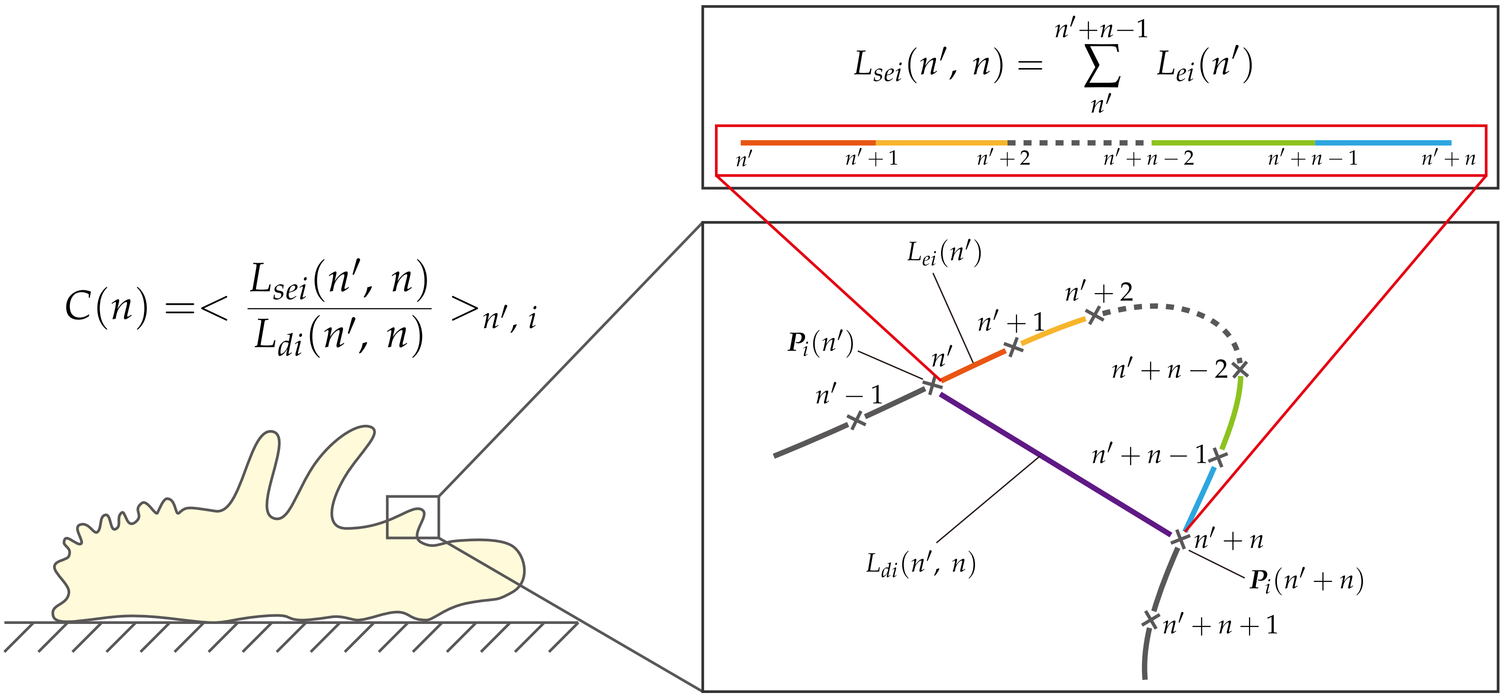


**Fig. S1.** Methods for analyzing the complexity of the cell membrane structure (see Materials & Methods for details).

Biology Open • Supplementary information

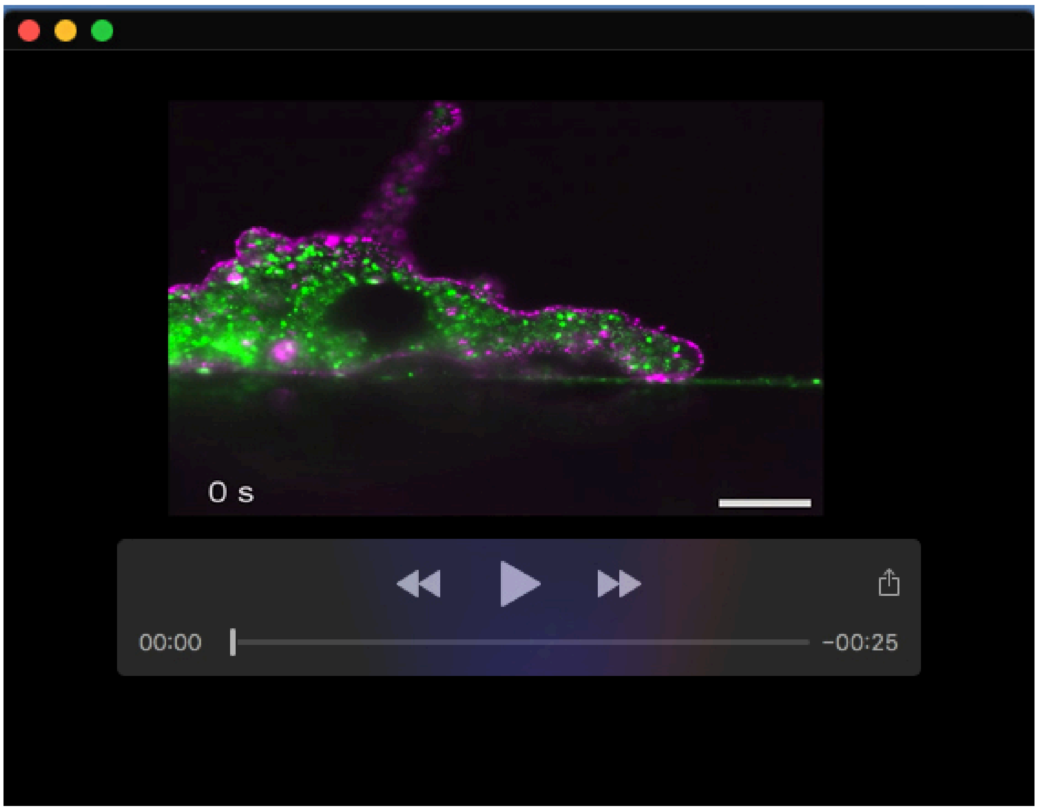


**Movie 1. Movement of the cell membrane and cytoplasm of *A. proteus* observed by light-sheet microscopy**

Magenta indicates the cell membrane, and green indicates mitochondria. The video playback speed is three times faster than the actual speed. The scale bar represents 30 µm.

Biology Open • Supplementary information

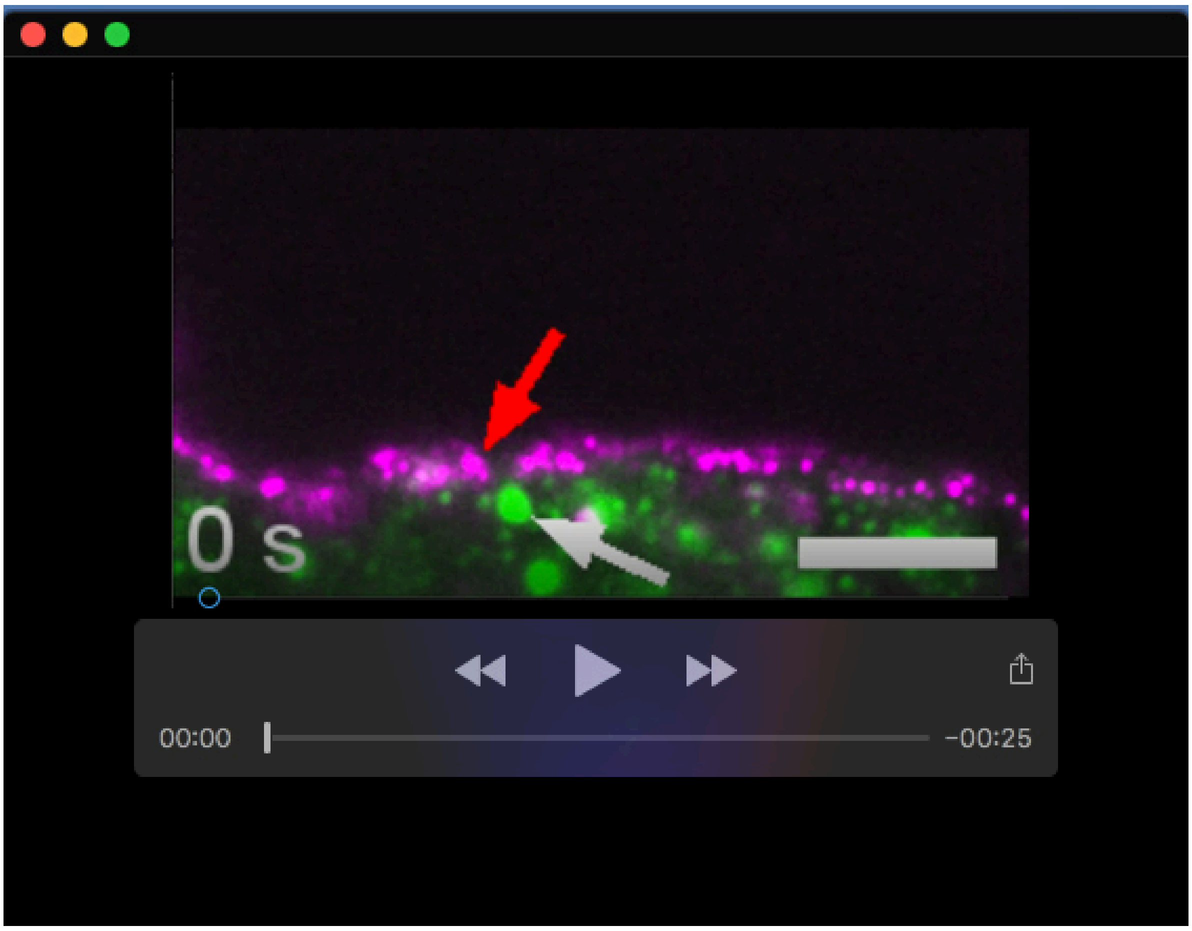


**Movie 2. Movement of the cell membrane and cytoplasm in the dorsal anterior region of *A. proteus***

Mitochondria (white arrows) in the cytoplasmic gel are almost stationary relative to the substrate, while the cell membrane (red arrows) moves in the direction of cell movement. The video playback speed is three times faster than the actual speed. The scale bar represents 10 µm.

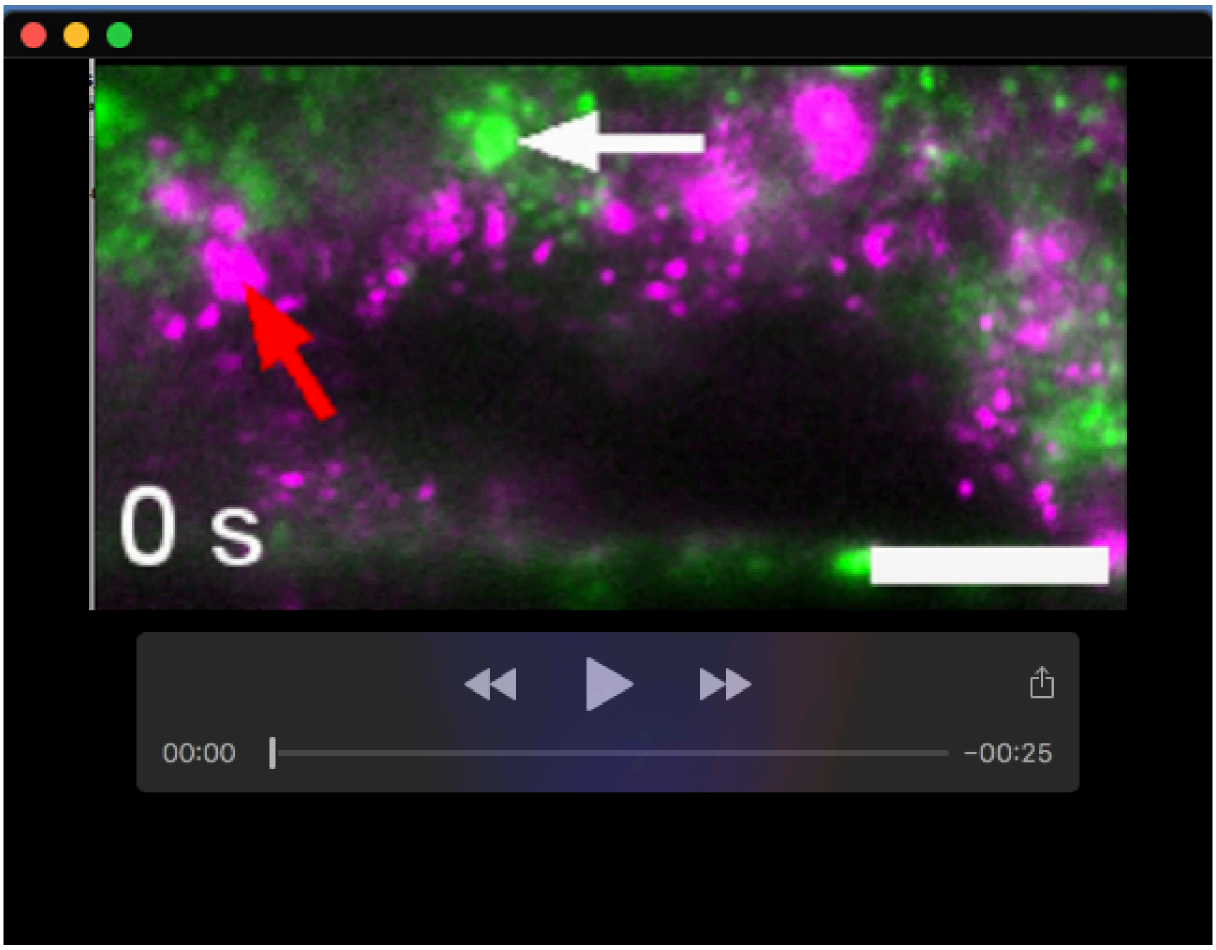


**Movie 3. Movement of the cell membrane and cytoplasm in the anterior ventral region of *A. proteus***

As in the dorsal anterior region, the mitochondria (white arrows) in the cytoplasmic gel were stationary relative to the substrate, while the plasma membrane (red arrows) moved in the direction of cell movement in the anterior ventral region. The video playback speed is three times the actual speed. The scale bar represents 10 µm.

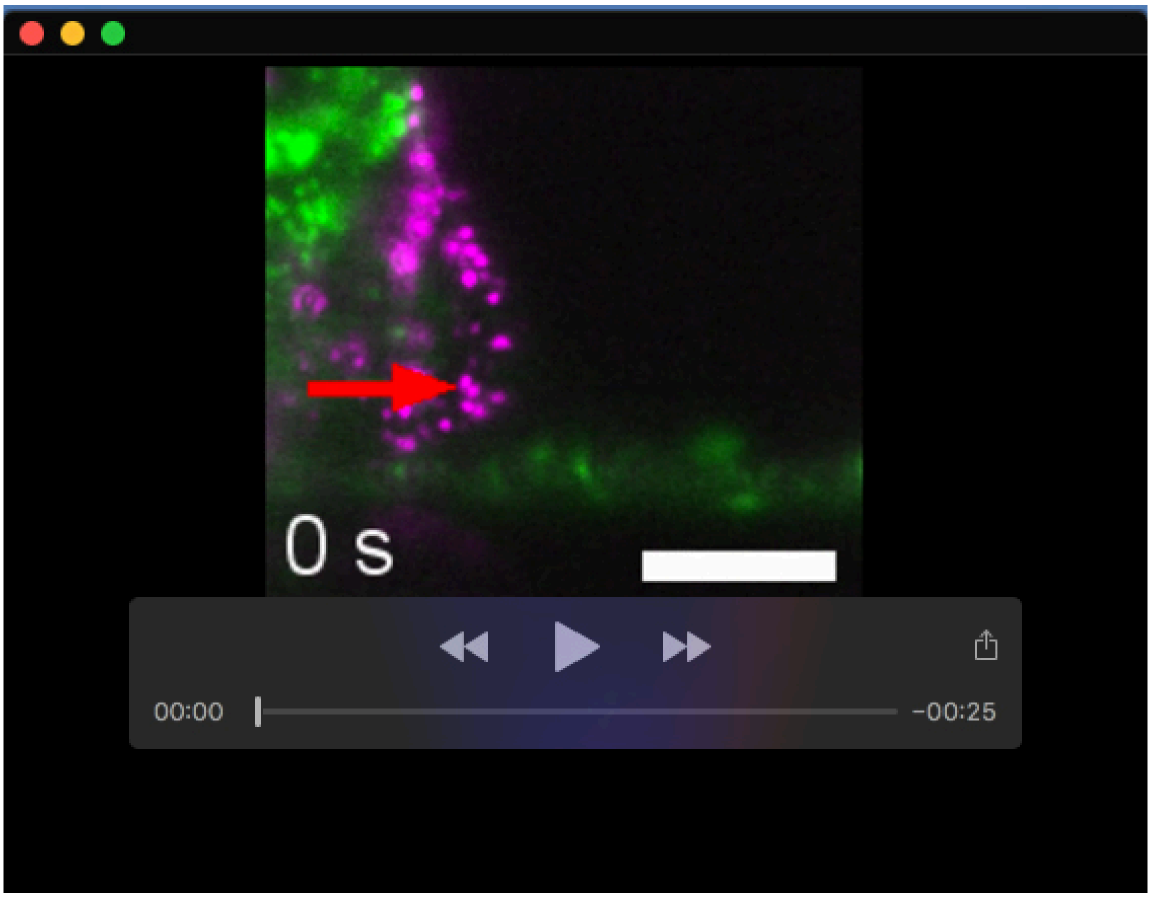


**Movie 4. Movement of the cell membrane and cytoplasm at the anterior end of *A. proteus***

The cell membrane (red arrow) was moving in the direction of cell movement. On the other hand, mitochondria in the cytoplasm (green) were difficult to trace due to their fast activity. The video playback speed is three times the actual speed. The scale bar represents 10 µm.

Biology Open • Supplementary information

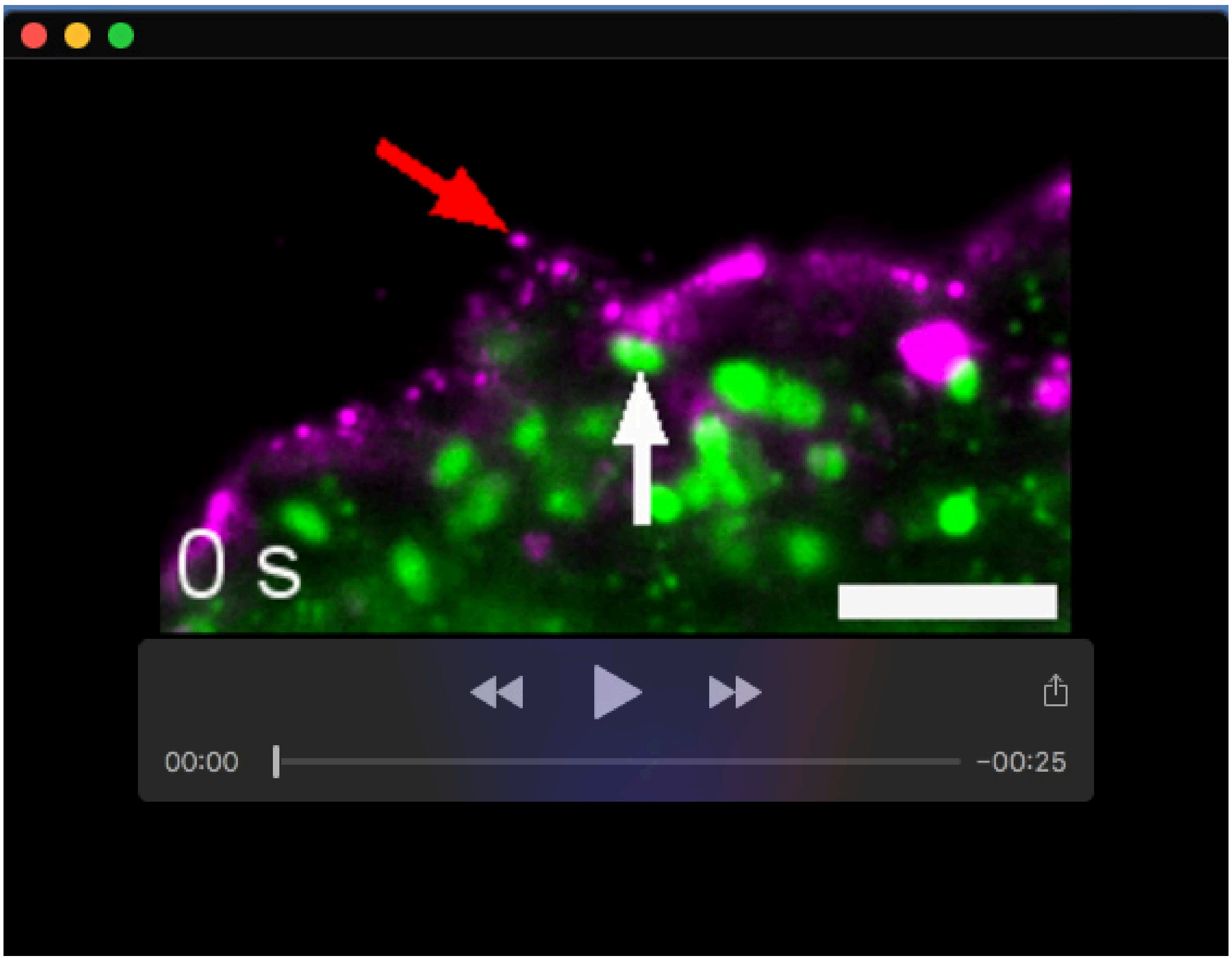


**Movie 5. Movement of the cell membrane and cytoplasm in the dorsal posterior region of *A. proteus***

Unlike other regions, the cell membrane (red arrow) and mitochondria in the cytoplasmic gel (white arrow) moved very slowly in the direction of cell movement. The video playback speed is three times the actual speed. The scale bar represents 10 µm.

Biology Open • Supplementary information

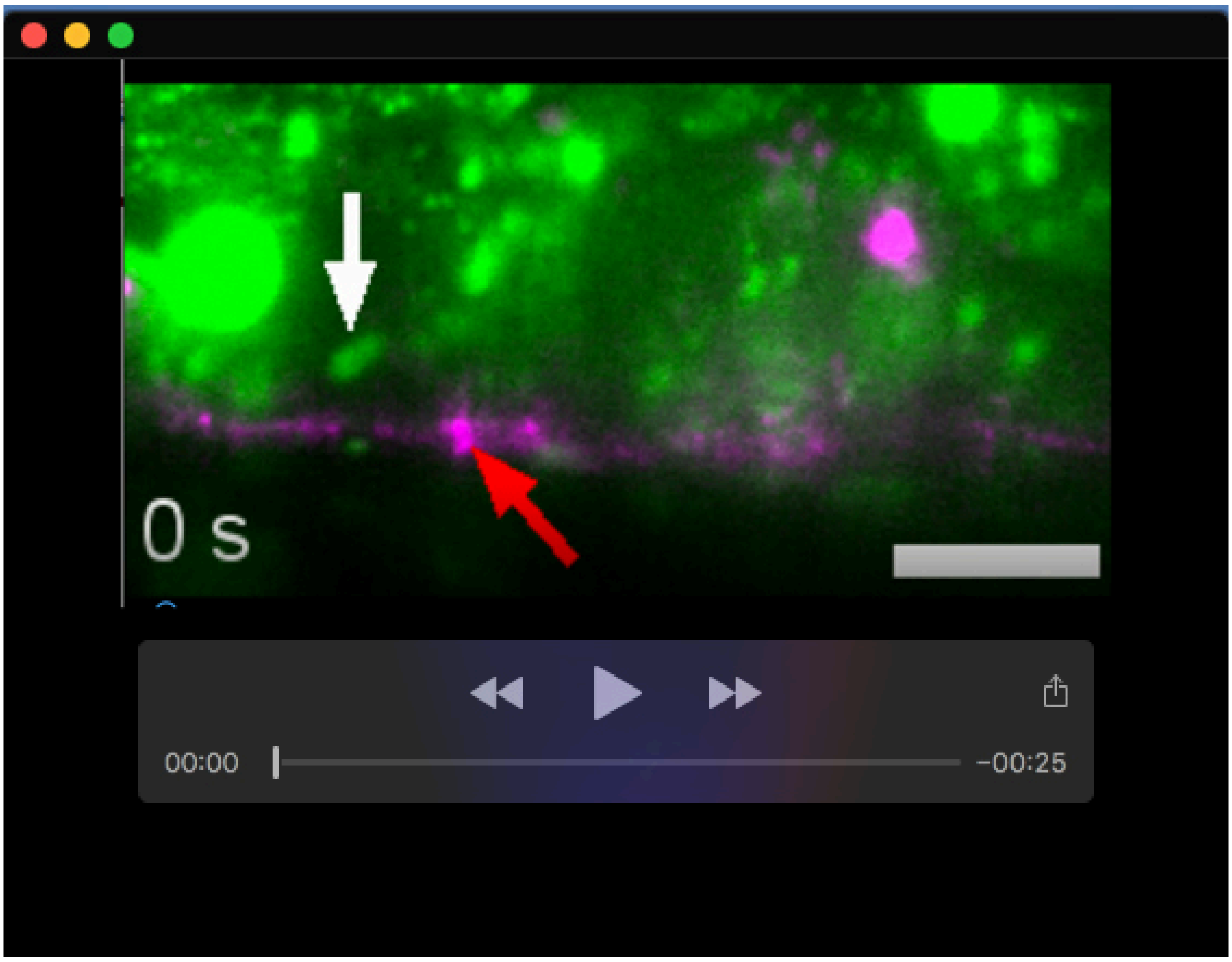


**Movie 6. Movement of the cell membrane and cytoplasm in the ventral posterior region of *A. proteus***

The cell membrane (red arrows) moved significantly in the direction of movement, while the mitochondria in the cytoplasmic gel (white arrows) moved slightly in the direction of cell movement. The video playback speed is three times the actual speed. The scale bar represents 10 µm.

Biology Open • Supplementary information

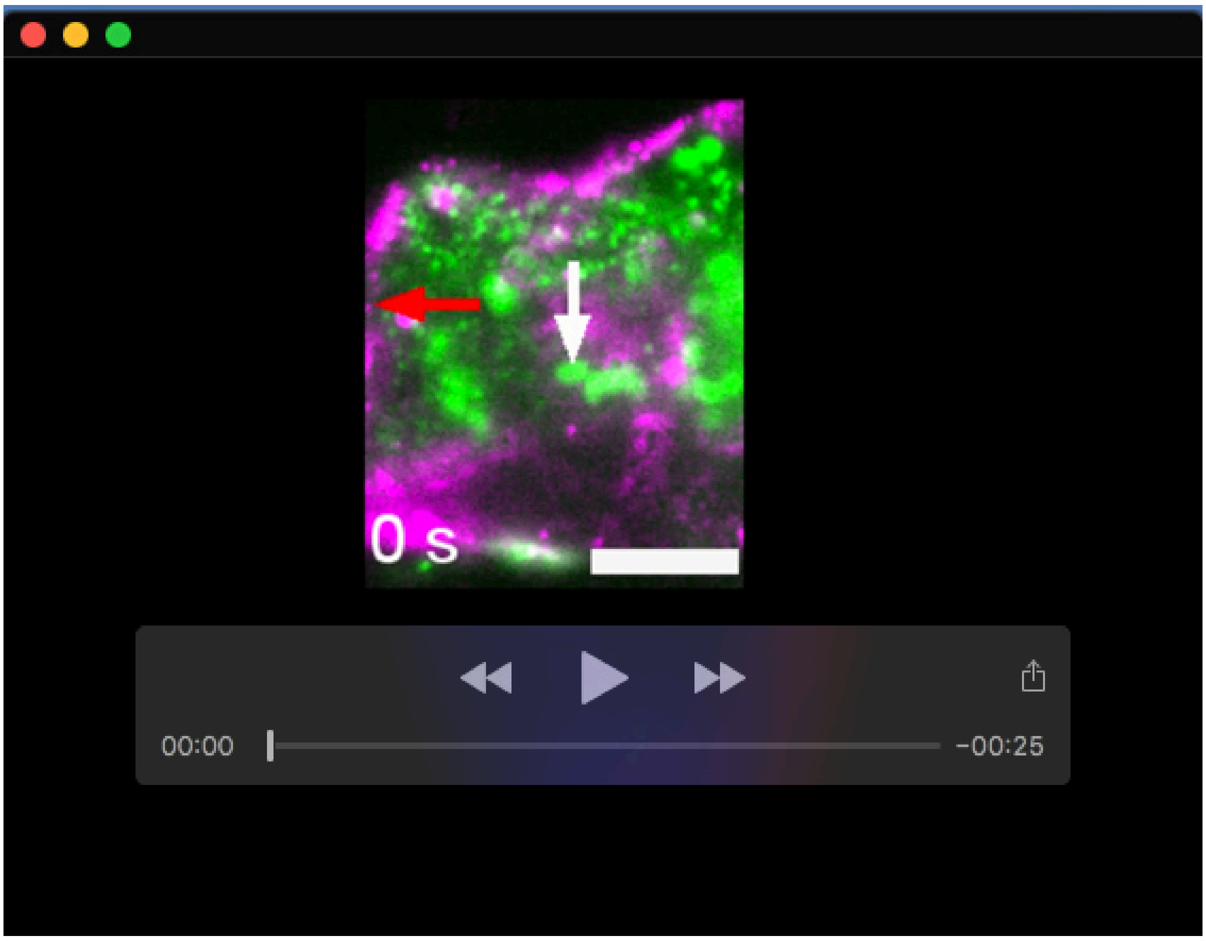


**Movie 7. Movement of the cell membrane and cytoplasm at the posterior end of *A. proteus***

As in the dorsal posterior region, the plasma membrane (red arrow) and mitochondria in the cytoplasmic gel (white arrow) exhibited slow motion. The video playback speed is three times the actual speed. The scale bar represents 10 µm.
